# Evaluation of the kitchen microbiome and food safety behaviors of predominantly low-income families

**DOI:** 10.3389/fmicb.2022.987925

**Published:** 2022-09-29

**Authors:** Christina K. Carstens, Joelle K. Salazar, Shreela V. Sharma, Wenyaw Chan, Charles Darkoh

**Affiliations:** ^1^Department of Epidemiology, Human Genetics and Environmental Sciences, School of Public Health, University of Texas Health Science Center, Houston, TX, United States; ^2^Division of Food Processing Science and Technology, U.S. Food and Drug Administration, Bedford Park, IL, United States; ^3^Department of Biostatistics and Data Science, School of Public Health, University of Texas Health Science Center, Houston, TX, United States; ^4^Microbiology and Infectious Diseases Program, University of Texas MD Anderson Cancer Center UTHealth Graduate School of Biomedical Sciences, Houston, TX, United States

**Keywords:** metataxonomics, indoor built environment, food safety, sanitation, consumer behavior, foodborne illness, diarrheal disease, demographic disparities

## Abstract

Bacterial pathogens in the domestic environment present a risk to residents, particularly among susceptible populations. However, the impact of consumer demographic characteristics and food handling methods on kitchen microbiomes is not fully understood. The domestic kitchen bacterial communities of ten predominantly low-income families in Houston, TX, were assessed in conjunction with a cross-sectional food safety survey to evaluate differences in household and surface-specific microbiomes and bacterial foodborne pathogen presence. Three kitchen surfaces within each household, including the sink drain, the refrigerator handle, and the counter, were environmentally sampled and metataxonomically evaluated via targeted 16S rRNA sequencing. Disposable dish sponges were also acquired and examined. Results indicated that alpha diversity did not vary by the households, sampling locations, or demographic characteristics evaluated. Significant differences in beta diversity were observed among the bacterial communities of five pairs of households and between refrigerator handle and disposable dish sponge microbiomes. A total of 89 unique bacterial foodborne pathogens were identified across surface types. Each household contained at least one contaminated surface, and the most common bacterial foodborne pathogens identified were *Escherichia coli*, *Staphylococcus aureus*, and *Klebsiella pneumoniae*. All parents reported washing their hands before meal preparation, washing fresh fruits and vegetables, and washing cutting boards with soap after use to prepare raw animal proteins. Gaps in food safety behaviors identified included a lack of serious concern for food contamination with germs and inappropriate handwashing, food handling, and cleaning behaviors. The number of unique bacterial foodborne pathogens identified within households was significantly higher among households whose respondent parent reported that they did not consider food contamination with germs to be a serious food safety problem (median: 41.0 species) compared to households whose respondent parent did consider food contamination to be a serious food safety problem (median: 3.0 species; *p* value = 0.0218). These results demonstrate that domestic kitchen taxonomic abundance profiles vary according to household and surface type. Data suggest that low-income consumers may be at risk of foodborne pathogen exposure from contaminated home kitchen surfaces, and that food safety attitudes may directly contribute to this hazard.

## Introduction

Foodborne infections are generally sporadic and underreported (Scallan et al., 2011b); however, an estimated 47.8 million episodes of foodborne illness occur in the United States annually (Scallan et al., 2011a). Bacteria are the primary cause of hospitalizations and deaths attributed to known foodborne pathogens (64%, respectively; Scallan et al., 2011b). Nontyphoidal *Salmonella* spp. (1,027,561 cases) are responsible for the most annual foodborne illnesses associated with bacterial etiologies, followed closely by *Clostridium perfringens* (965,958 cases) and *Campylobacter* spp. (845,024 cases). Treatment in the U.S. for a single case of foodborne illness averages about $1,068, which places an approximate $77.7 billion burden on the healthcare system each year (Scharff, 2012). Of known bacterial pathogens, nontyphoidal *Salmonella* spp. ($11.391 billion), *Campylobacter* spp. ($6.879 billion), and *Listeria monocytogenes* ($2.040 billion) cause the largest economic burden based on the cost of treatment.

Approximately 20% of the U.S. population is considered susceptible to foodborne illness due to factors such as age or certain medical treatments (Gerba et al., 1996). Low-income and racial-ethnic minority population groups, including African American, Asian American, and Hispanic communities in the U.S. also experience higher incidence rates of several foodborne infections than European Americans, yet the mechanisms that contribute to these disparities are not fully understood (Quinlan, 2013). Although most common foodborne infections are preventable with adequate personal hygiene, cleaning, food preparation, and cooking methods (Medeiros et al., 2001), the consumption of food prepared in the home is considered a leading cause of foodborne disease outbreaks (Odeyemi and Sani, 2016). Consequently, consumers are considered the last line of defense against foodborne infections. Various unsafe food handling practices that may promote cross-contamination, such as washing raw poultry before preparation, appear to be unique to or more prevalent among racial and ethnic minority groups (Henley et al., 2012, 2015), which may contribute to the elevated risk of home-acquired foodborne illness among these communities.

The past two decades have seen an increase in the proportion of outbreaks associated with food prepared in private residences. According to the U.S. Centers for Disease Control and Prevention (CDC), the percentage of foodborne disease outbreaks attributable to home-prepared food has increased from 9% between 1998 and 2008 (CDC, 2013) to 12% between 2009 and 2015 (CDC, 2018). Data available through the National Outbreak Reporting System also demonstrates an increase in the annual number of home-attributed foodborne outbreaks in the latter part of the past decade (CDC, 2022). Yet, a recent survey conducted by the U.S. Food and Drug Administration (FDA; U.S. Food and Drug Administration, 2021) has revealed that most consumers in the U.S. do not perceive home-prepared foods as risky and instead consider restaurant-prepared foods the more likely source of foodborne illness (FDA, 2021). Moreover, 95% of surveyed U.S. consumers also agreed that they know how to cook food safely (FDA, 2011).

Bacteria can enter the domestic environment via numerous routes, including human skin, water, and food products (Flores et al., 2013). Bacterial communities within homes are known to contain human pathogens and multi-drug resistant species, and kitchens harbor the highest levels of bacterial contamination (Marshall et al., 2012; Borrusso and Quinlan, 2017b). In the built environment, areas to which bacteria localize include sink drains, dish sponges or cloths, and food contact surfaces, such as counters. Environmental contamination with bacterial foodborne pathogens and inadequate food safety measures can lead to cross-contamination during food preparation, which increases the risk of home-acquired illness. For example, fresh produce, which is commonly consumed raw and frequently implicated in multistate outbreaks of foodborne illness in the U.S. (Carstens et al., 2019), can transport pathogens into home kitchens and lead to bacterial cross-contamination during food preparation. As cross-contamination frequently contributes to foodborne disease outbreaks, the minimization of cross-contamination events is a critical public health concern (Evans et al., 1998).

Although domestic kitchen ecology has been examined previously, restrictions associated with methodological approaches have presented challenges. Specifically, studies that have used culture-based techniques (Josephson et al., 1997; Ojima et al., 2002; Borrusso and Quinlan, 2017b) did not evaluate the complete microbiome and relied on bacterial culturability. Studies that have used culture-independent approaches, such as metataxonomic sequencing, did not obtain species level taxonomic identifications (Dunn et al., 2013; Flores et al., 2013; Lax et al., 2014), which are necessary for the identification of foodborne pathogens. In various cases, past studies of kitchen ecology did not present detailed demographic on information participants (Josephson et al., 1997; Ojima et al., 2002; Dunn et al., 2013; Flores et al., 2013; Lax et al., 2014), which is necessary to identify factors that may contribute to the elevated incidence of foodborne illness among low-income and racial-ethnic minority communities. As a result, the goal of this study was to evaluate potential associations between parent demographic and behavioral characteristics and bacterial foodborne pathogen presence in the domestic kitchens of predominantly low-income families using targeted 16S rRNA sequencing. Specifically, this study aimed to (1) assess the taxonomic diversity and distribution of kitchen bacterial communities, (2) evaluate differences in household and surface-specific bacterial microbiomes, (3) ascertain the prevalence of bacterial foodborne pathogen presence, and (4) examine bacterial foodborne pathogen presence according to self-reported food handling behaviors.

## Materials and methods

### Participants and enrollment

Recruitment, enrollment, and sample collection took place over six months, from January to June 2021. Parents of children currently enrolled in elementary schools within Houston Independent School District (HISD) were recruited via partnership with Brighter Bites. Brighter Bites is a nonprofit 16-week intervention that provides fresh produce and nutrition education in schools that serve predominantly low-income families, as indicated by a minimum of 75% of the student body receiving free or reduced-price lunch (Title 1; Sharma et al., 2016). Recruitment was conducted using a convenience sampling approach via the dissemination of electronic and physical flyers advertising the study in English and Spanish. Flyers were emailed to parents and posted to online classroom message boards by Brighter Bites staff. Physical flyers were also handed out at in-person food distribution events hosted by Brighter Bites. Inclusion criteria for participation in the current study included individuals ≥ 18 years old who were the parent or guardian of at least one child enrolled in any HISD elementary school. Study requirements included completion of an online survey and in-home environmental sampling appointment. Electronic consent was collected before the online survey, and written consent was collected before initiating in-home sampling. A total of ten respondent parents representing ten households were enrolled in the study and completed the study requirements. This study was approved by the University of Texas Health Science Center Committee for the Protection of Human Subjects (reference number: HSC-SPH-20-1155; approval date: November 18, 2020).

### Survey instrument

The survey instrument has been described previously (Carstens et al., 2022) and was used to evaluate each respondent parent’s demographic characteristics, food safety attitude, handwashing, kitchen cleaning, and food handling behaviors. Survey questions were modified from the U.S. Food and Drug Administration’s Food Safety Survey Kwon et al. (2008) and (FDA, 2021) and were in multiple-choice format. The survey was administered electronically using Qualtrics online survey platform (Qualtrics, 2020; version January–May, 2021; Qualtrics, Provo, UT, United States) and was available in both English and Spanish. A total of 20 food safety-related behavioral outcomes were evaluated via the survey and the total time commitment required to complete the questionnaire was approximately 10 minutes.

Food safety behavioral outcomes were considered appropriate or inappropriate based on methods recommended by the U.S. FDA or the United States Department of Agriculture (USDA). Appropriate defrosting methods included “in the refrigerator,” while inappropriate methods included “on the counter,” “in sink of water,” and “under running water” as thawing time and water temperature were not evaluated (USDA, 2013). “Washing with soap” was considered the appropriate response for handling a cutting board after contact with raw animal proteins and before reuse to prepare other food to be eaten raw for the same meal (USDA, 2017). Inappropriate contaminated cutting board handling included “rinsing” or “wiping.” For leafy greens and melons, “rubbing under running water” was considered the appropriate response, while inappropriate responses included “hold under running water, without rubbing,” “soak in a container of water,” and “use a cleaner to wash” (FDA, 2021). The use of sponges and dish cloths to clean the kitchen counter was considered inappropriate for this analysis as these tools are known to contain fecal coliform and foodborne pathogen contamination (Borrusso and Quinlan, 2017a).

### Sampling locations and processing

Environmental surface samples were collected from each household’s kitchen using pre-moistened PUR-Blue swabs in Dey-Engley Neutralizing Broth (DE; World Bioproducts, LLC, Libertyville, IL, United States). Sampling locations were not revealed to participants in advance and included (1) the refrigerator handle (front and back), (2) the sink drain, and (3) a 4-in × 4-in area next to the kitchen sink. The sink drain was sampled underneath the drain trap or above the disposal entrance if a garbage disposal was present. Disposable dish sponges were also collected. After collection, swabs were placed in sterile tubes with 2 ml 1 × phosphate-buffered saline (PBS), and disposable dish sponges were placed in sterile bags with 50 ml PBS. All samples were stored on ice for no more than 1 h before processing. Tubes with swabs were vortexed for 1 min, while bags with dish sponges were massaged by hand for 1 min. Sample aliquots (1 ml) were supplemented with glycerol at 20% and stored at −20°C until further processing.

### DNA extraction and PCR amplification of 16S rRNA genes

DNA was extracted in duplicate using a 100 μl starting volume from each of the four sample types collected from the ten households (*n* = 80) using the DNeasy Blood and Tissue Kit (Qiagen Inc., Germantown, MA, United States). DNA was quantified using the Qubit dsDNA HS Assay Kit (Invitrogen, Carlsbad, CA). Extracted DNA products were stored at −20°C before PCR assays. For PCR reactions, four primer pairs were used to target the V1–V3 region of the bacterial 16S rRNA gene as described in Salazar et al. (2018) and were distributed randomly and equally among all DNA samples. PCR products were visualized using agarose gel electrophoresis and quantified using the Qubit dsDNA HS Assay kit (Invitrogen). PCR products were purified using AMPure XP beads (Beckman-Coulter, Indianapolis IN, United States) and stored at −20°C.

### Library construction and sequencing

The Nextera XT Kit (Illumina, San Diego, CA, United States) was used to index 16S rRNA gene fragments as previously described (Salazar et al., 2018). Indexed PCR products were quantified using the Qubit dsDNA HS Assay kit (Invitrogen) and normalized to 2 nM in 10 mM Tris–HCL, 0.1% Tween 20, pH 8.5. Normalized, indexed samples were pooled, diluted to 10 pM, spiked with 10% of 12.5 pM PhiX, and sequenced using 500 cycles of MiSeq version 2 chemistry (Illumina).

### Data analysis

Raw, paired-end reads were merged and filtered based on quality (Q30, 99.9% accuracy) and length (minimum 300 base pairs). Bacterial taxonomic classifications at the family, genus, and species level were obtained using Kraken2 (Wood et al., 2019) and comparison with the Ribosomal Database Project (RDP; Cole et al., 2014) and the National Center for Biotechnology Information (NCBI; O’Leary et al., 2016) 16S databases. The relative abundance of each taxon was estimated using Bracken (Lu et al., 2017). Any sample that contained a bacterium known to cause foodborne illness (FDA, 2012) was considered positive for a bacterial foodborne pathogen. Although not all members of *Escherichia coli* are pathogenic to humans, when this species was identified, it was included as a possible foodborne pathogen as these species were of unknown serotype.

Summary statistics, including frequencies and percentages, were generated for demographic characteristics, food safety behaviors, and bacterial foodborne pathogen presence across sampling locations and households. Three pathogen-related outcomes were examined, including the numbers of genera with relative abundances > 5%, bacterial foodborne pathogen-containing genera with relative abundance > 5%, and unique bacterial foodborne pathogens. Each of these outcomes were evaluated for normality using visual inspection and the Shapiro–Wilke test. Medians and ranges were computed for the numbers of genera with relative abundances > 5%, bacterial foodborne pathogen-containing genera with relative abundance > 5%, and unique bacterial foodborne pathogens, which were statistically compared by sampling location using the Kruskal–Wallis test. The number of foodborne pathogen-containing genera with relative abundance > 5% and unique foodborne pathogens were further statistically compared by demographic characteristics and food safety behaviors using the Mann–Whitney *U*-test. The number of sampling locations positive for foodborne pathogen contamination per household was summarized with medians and ranges by demographic group and food safety behaviors and statistically compared using the Mann–Whitney *U*-test.

The alpha and beta diversity of identified bacterial communities was evaluated using parameters computed via the “vegan” package for R (Oksanen et al., 2013). Relative abundances were averaged between replicate samples and used to calculate measures of bacterial community diversity, including Shannon’s diversity index, Simpson’s index, and Simpson’s reciprocal index. The number of reads assigned to each taxon was averaged between replicate samples and used to calculate measures of taxonomic richness, including the number of observed taxonomic units and the Chao 1 index. Alpha diversity parameters were evaluated for normality using visual inspection and the Shapiro-Wilke test, summarized using medians and ranges by household, sampling location, and demographic group, and statistically compared using the Mann–Whitney *U*-test or the Kruskal–Wallis test. The beta diversity of each bacterial community was visualized via non-metric multidimensional scaling (NMDS) using the Bray–Curtis dissimilarity metric. Differences in bacterial community taxonomic abundance profiles across households, sampling locations, and demographic groups were statistically evaluated using pairwise permutational multivariate analysis of variance (PERMANOVA) tests and the Bonferroni correction computed via the “pairwiseAdonis” package for R (Martinez Arbizu, 2020).

All data analysis was conducted using the R language (RCoreTeam, 2017; version 3.6.3, R Foundation for Statistical Computing, Vienna, Austria) and RStudio software (RStudioTeam, 2020; version 4.1.2; RStudio, Inc., Boston, MA, United States). Data visualization was also conducted using Tableau software (version 2020.2.4, Tableau Software, Inc., Seattle, WA, United States; Tableau, 2022). A *p* value < 0.05 was considered statistically significant.

## Results

A total of ten parents of elementary-aged children participated in both the survey and home kitchen environmental sampling. Study participants were primarily female (60.0%), Hispanic (60.0%), had high school or higher education (70.0%), and prepared animal proteins (i.e., poultry, meat, or seafood) from a raw state (77.8%; Supplemental Table 1). Most households primarily spoke a language other than English (70.0%), did not include a resident ≥ 65 years of age (90.0%) or a pregnant resident (100.0%), and almost half owned a pet (40.0%). The internet was the most commonly reported primary source of food safety information (40.0%), followed by the television (30.0%), school (20.0%), and work (10.0%).

Environmental samples were collected from ten households to evaluate bacterial community diversity and foodborne pathogen presence within the domestic kitchens of predominantly low-income families. A total of 80 samples from three kitchen surface types, including sink drains, refrigerator handles, and counters, along with disposable dish sponges, were characterized metataxonomically via 16S rRNA sequencing.

### Alpha diversity

**Figure 1:**
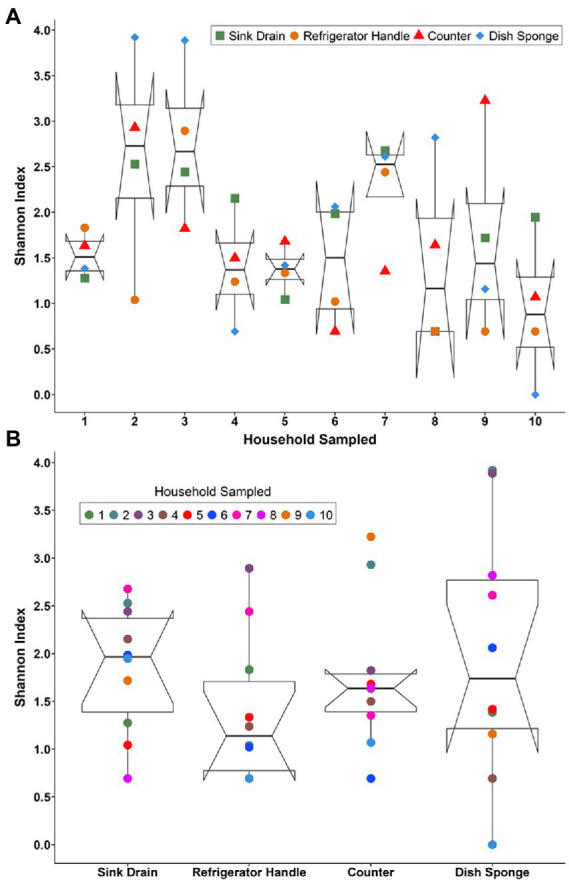
The alpha diversity of the microbial communities detected among four kitchen sampling locations within predominantly low-income households (*n* = 10) by household **(A)** and sampling location **(B)** as represented by Shannon’s diversity index, Houston, Texas, 2021. Shannon’s diversity index parameter computations were conducted using the averaged relative abundances of taxa between replicate samples.

Bacterial community alpha diversity, as represented by Shannon’s diversity index, was not significantly different across households (Figure 1A) or sampling locations (Figure 1B). However, a large degree of variation in diversity was noted across sampling locations and households. Median Shannon’s diversity index values per household ranged from 0.88 to 2.73. Sink drains were the most diverse kitchen location sampled, followed by dish sponges, counters, and refrigerator handles (Supplemental Tables 2–5). The number of operational taxonomic units (OTUs) at each sampling location also did not vary significantly according to household or sampling location (Supplemental Tables 2–5). The median number of OTUs per household ranged from 3.0 to 158.5 OTUs. Across households, a wide range of OTU counts was observed for each sampling location. Although sink drains were the most diverse location sampled, dish sponges had the highest number of OTUs identified, ranging from 1 to 646. Refrigerator handles had the lowest number of OTUs identified, ranging from 2 to 44. The alpha diversity of household bacterial communities, as represented by Shannon’s diversity index (Supplemental Figure 1) and the number of observed OTUs, was not observed to vary significantly according to the demographic variables evaluated, including respondent parents’ age, education, employment status, primary household language, previous food handling employment experience, pet ownership, race/ethnicity, or sex.

### Relative abundance of bacterial taxa > 5%

**Figure 2:**
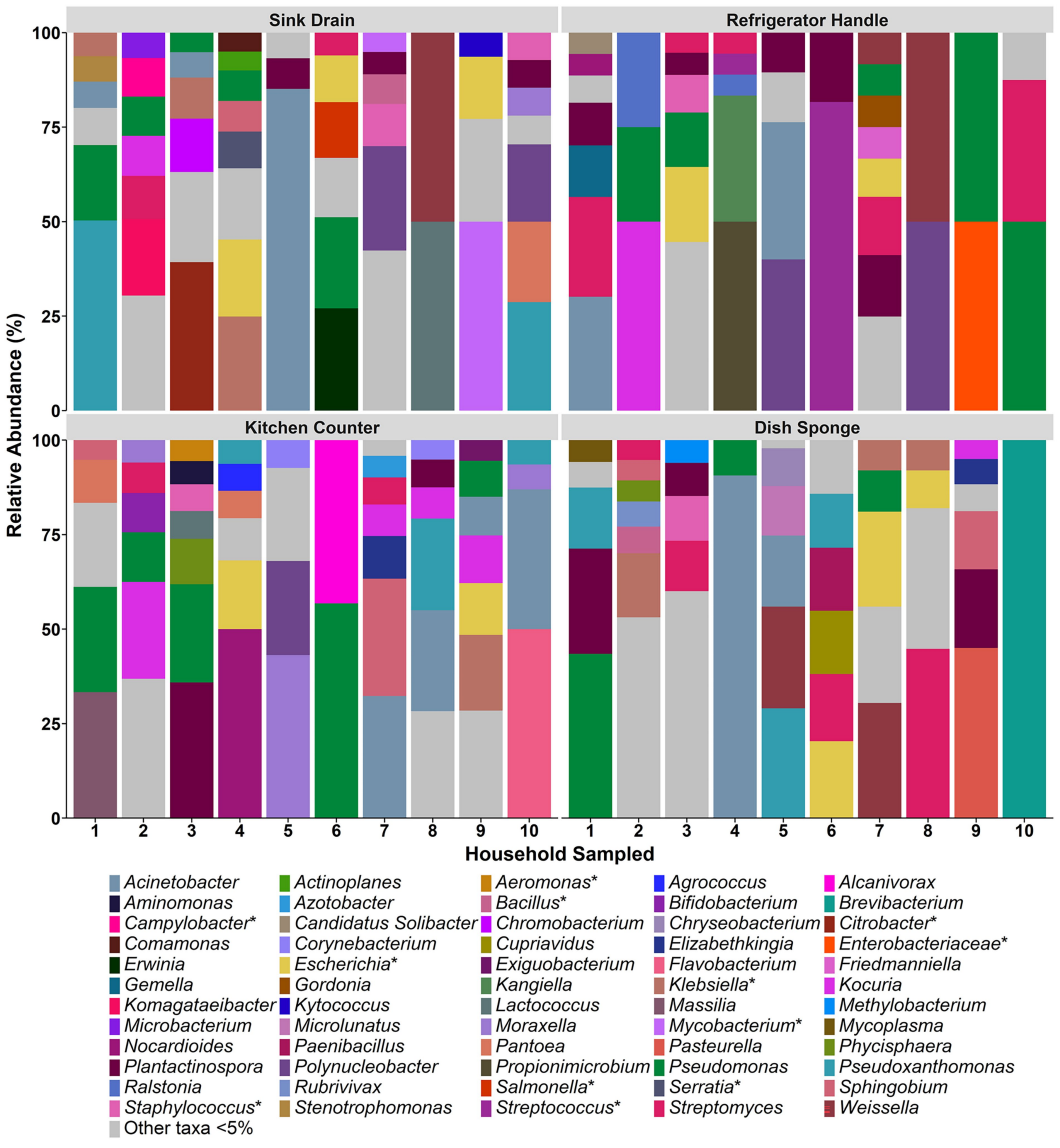
The relative abundance of bacterial taxa detected at the genus level among four kitchen sampling locations within predominantly low-income households (*n* = 10), Houston, Texas, 2021. Bars represent averaged relative abundances of taxa between replicate samples. Pathogen-containing genera are indicated with an asterisk.

The relative abundances of identified bacterial taxa >5% at both the family (Supplemental Figure 2) and genus level (Figure 2) in each microbiome were averaged across replicate samples and evaluated according to household and sampling location. Across all kitchen surfaces and households, 60 unique taxa were identified. Of these taxa, 59 were at the genus level, and the remaining taxon was at the family level (unassigned members of the *Enterobacteriaceae* family). The median number of taxonomic identifications per household ranged from 9.0 to 17.0, and no one taxon appeared in more than three of the four sampling locations per household. *Pseudomonas* was the most frequently occurring taxon in seven of the ten households evaluated, followed by *Escherichia* (4 households) and *Streptomyces* (3 households).

Across sample types, sink drains and kitchen counters contained the highest median number of taxa (5.0 taxa, respectively), ranging from 2 to 7 taxa per location. The median number of taxa per sample type was second highest for dish sponges (4.0 taxa), ranging from 1 to 6 taxa per sponge, and lowest for refrigerator handles (3.0 taxa), ranging from 2 to 7 taxa per handle. No significant difference was detected in the number of taxa according to sample type. A total of 29 taxa were identified across the sink drains evaluated, and *Pseudomonas* was the most common (5 of 10 sink drains). Across kitchen counters, 27 taxa were identified. *Pseudomonas* was also the most frequently identified taxon at this location (5 of 10 kitchen counters), followed by *Acinetobacter* and *Kocuria* (4 of 10 kitchen counters, respectively). A total of 23 taxa were identified among dish sponges, of which *Streptomyces* was the most frequently identified (4 of 10 dish sponges). Among refrigerator handles, 19 taxa were identified, and *Plantactinospora*, *Pseudomonas*, and *Streptomyces* were the most common taxa identified across the ten handles evaluated (5 refrigerator handles, respectively).

### Bacterial foodborne pathogen presence

Foodborne pathogens within each domestic kitchen were evaluated according to the number of unique foodborne pathogen containing-genera with > 5% relative abundance and the number of unique foodborne pathogens identified at each sampling location and within each household. A total of 11 foodborne pathogen-containing genera and 89 foodborne pathogens were identified across all households and sampling locations. Across the 40 environmental samples, the most frequent pathogen-containing genera identified > 5% were *Escherichia* (10 samples), *Klebsiella* (7 samples), and *Staphylococcus* (5 samples). The most common foodborne pathogens identified, including those < 5%, were *E. coli* (15 samples), *Staphylococcus aureus* (14 samples), *Klebsiella pneumoniae* (12 samples), and *Serratia marcescens* (10 samples).

**Figure 3:**
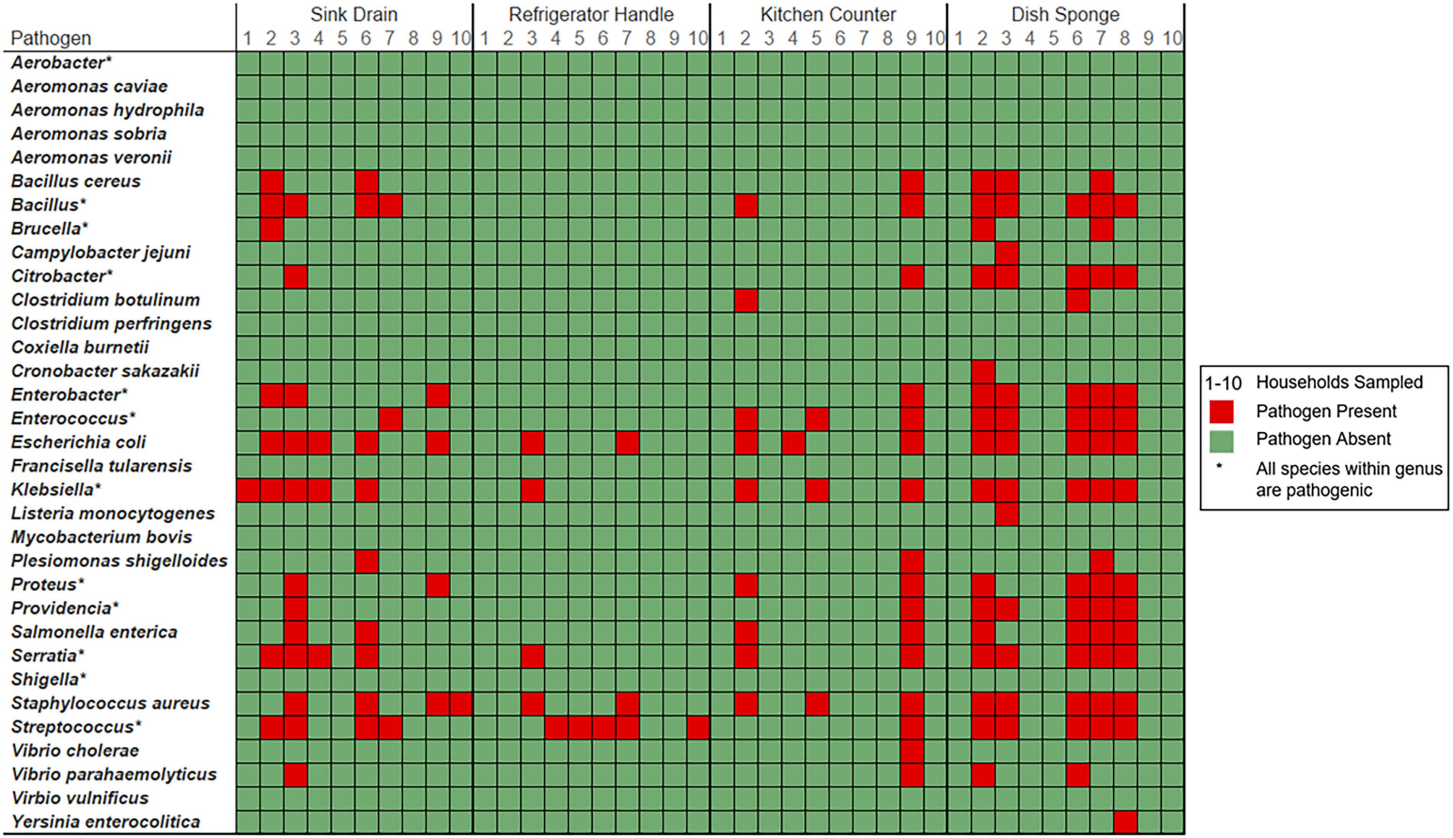
Foodborne pathogen presence detected among four kitchen sampling locations within predominantly low-income households (*n* = 10) according to household and sampling location, Houston, Texas, 2021. Red color indicates that the respective sample contained a bacterium known to cause foodborne illness (FDA, 2012). Green color indicates that bacterial foodborne pathogens of interest were not identified within the sample. Pathogen-containing genera are indicated with an asterisk. The number of unique species level identifications per pathogen containing genera included: *Bacillus* (23), *Brucella* (2), *Citrobacter* (6), *Enterobacter* (6), *Enterococcus* (6), *Klebsiella* (6), *Proteus* (2), *Providencia* (2), *Serratia* (6), and *Streptococcus* (14).

Foodborne pathogen contamination was not identified at more than three out of four sampling locations per household (Figure 3). The number of foodborne pathogen-containing genera identified per household ranged from 1 to 5 taxa (median: 3.0 taxa), while the number of foodborne pathogens identified per household ranged from 1 to 51 species (median: 25.0 species). Across sampling locations, *Escherichia* was the most frequently identified foodborne pathogen-containing genus within five households, followed by *Klebsiella* (3 households) and *Staphylococcus* (2 households). *E. coli* and *S. aureus* were the most frequently identified foodborne pathogens across sampling locations in six households, followed by *K. pneumoniae* (5 households). No one foodborne pathogen was identified in more than three of the four sampling locations per household.

Among the four sampling locations, sink drains were most frequently positive for foodborne pathogen contamination (8 households), followed by refrigerator handle (6 households), dish sponge (5 households), and counter (4 households). The median number of foodborne pathogens identified at each sampling location was highest among dish sponges (median: 7.5 species) and ranged from 0 to 34 species per sponge. A range of 0 to 18 foodborne pathogens was identified per sink drain (median: 3.0). A similarly low median number of foodborne pathogens was identified among refrigerator handles and counters (median: 0.0 species, respectively); however, a range of 0 to 5 species was identified across refrigerator handles, while a range of 0 to 51 species was identified across counters. No significant differences in the number of foodborne pathogens or the number of foodborne pathogen-containing genera were detected across the four sampling locations.

Four foodborne pathogen-containing genera were identified across all dish sponges evaluated, and dish sponges contained the highest number of foodborne pathogens identified across sampling locations of the same type (68 species). Most of the foodborne pathogens identified among sponges were unique to only one dish sponge (41 species). The most common foodborne pathogens identified among dish sponges included *E. coli*, *K. pneumoniae*, *Serratia marcescens*, and *S. aureus* (five sponges each). Four foodborne pathogen-containing genera and 52 foodborne pathogens were identified across all counters evaluated. *E. coli*, *K. pneumoniae*, and *S. aureus* were the most common foodborne pathogens identified among counters; however, most foodborne pathogens were only present on one of the ten counters sampled (44 out of 52 species). A total of nine unique pathogen-containing genera and 35 foodborne pathogens were identified across sink drains. *E. coli* was identified among half of the sink drains sampled (5 sink drains), while *Bacillus thuringiensis*, *K. pneumoniae*, and *S. aureus* were identified in four sink drains each. Most foodborne pathogens identified among sink drains were only present in one of the ten drains sampled (24 out of 35 species). Only three foodborne pathogen-containing genera and seven foodborne pathogens were identified among refrigerator handles. The most common pathogens identified among refrigerator handles included *E. coli*, *S. aureus*, and *Streptococcus mitis* (2 refrigerator handles each), while the remaining four pathogenic species identified were present on one refrigerator handle each.

### Beta diversity

**Figure 4:**
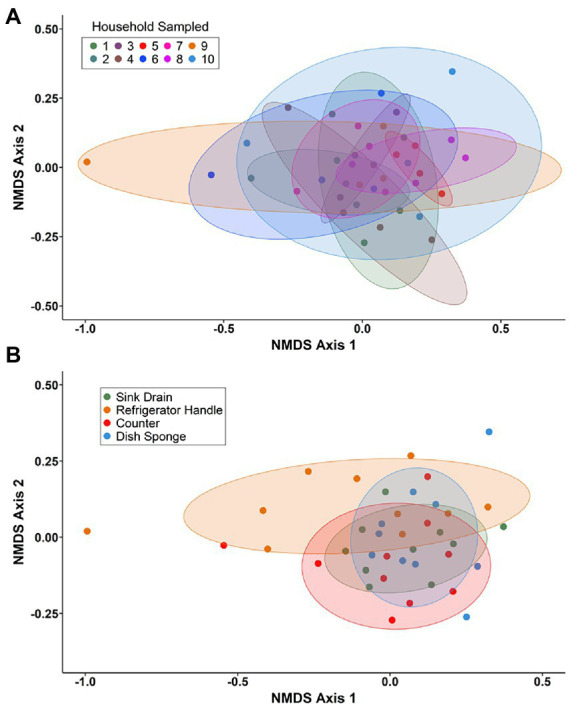
Non-metric multidimensional scaling (NMDS) of Bray–Curtis dissimilarity for bacterial communities present among four kitchen sampling locations within predominantly low-income households (*n* = 10) by household **(A)** and sampling location **(B)**, Houston, Texas, 2021. Beta diversity computations were conducted using the averaged relative abundances of taxa between replicate samples.

Non-metric multidimensional scaling (NMDS) using Bray–Curtis dissimilarity was utilized to visualize differences in bacterial communities by household (Figure 4A) and by sampling location (Figure 4B). Pairwise permutational multivariate analysis of variance (PERMANOVA) revealed significant differences in taxonomic abundance between the microbiomes of five pairs of houses (Table 1). Specifically, the microbiome of household 2 was significantly different from three households, and the microbiome of household 3 was significantly different from two households. Across the four sampling locations, a significant difference in taxonomic abundance was also observed between refrigerator handle and dish sponge microbiomes (*p* value = 0.0440; Table 2). No significant variation in beta diversity was observed according to the demographic characteristics evaluated.

**Table 1:** Predominantly low-income households (*n* = 10) with significantly different microbial community beta diversity as detected using pairwise permutational multivariate analysis of variance (PERMANOVA) and Bray–Curtis dissimilarity, Houston, Texas, 2021.

Comparison	Sum of squares^a^	*F* value^b^	*r*^2,^ ^c^	Adjusted *p* value^d^
House 1 vs. 2	0.6203	1.4972	0.1997	0.03996^*^
House 2 vs. 4	0.6405	1.4956	0.1995	0.01998^*^
House 2 vs. 5	0.7296	1.8827	0.2388	0.03297^*^
House 3 vs. 5	0.5626	1.4260	0.1920	0.04995^*^
House 3 vs. 10	0.5574	1.2673	0.1744	0.04895^*^

* Indicates a significant *p* value (*p* < 0.05).

a The sum of squares represents the total variation.

b The *F* value represents a ratio of the within-group dissimilarities and the between group dissimilarities.

c The coefficient of determination represents the percentage of the variance explained by the groups.

d *p* values were adjusted for multiple comparisons using the Bonferroni method.

**Table 2:** Pairwise permutational multivariate analysis of variance (PERMANOVA) of the microbial community beta diversity present among four kitchen sampling locations within predominantly low-income households (*n* = 10) using Bray–Curtis dissimilarity, Houston, Texas, 2021.

Comparison	Sum of squares^a^	*F* value^b^	*r*^2,^ ^c^	Adjusted *p* value^d^
Sink drain vs. refrigerator handle	0.5783	1.2963	0.0672	0.11190
Sink drain vs. kitchen counter	0.3320	0.7336	0.0392	0.93210
Sink drain vs. dish sponge	0.2914	0.6654	0.0357	0.94410
Refrigerator handle vs. kitchen counter	0.5260	1.1761	0.0613	0.21980
Refrigerator handle vs. dish sponge	0.6347	1.4670	0.0754	0.0440*
Kitchen counter vs. dish sponge	0.4282	0.9752	0.0514	0.49550

* Indicates a significant *p* value (*p* < 0.05).

a The sum of squares represents the total variation.

b The *F* value is a ratio that compares the within-group dissimilarities to the between group dissimilarities.

c The coefficient of determination represents the percentage of the variance explained by the groups.

d *p* values were adjusted for multiple comparisons using the Bonferroni method.

### Food safety behaviors

The food safety practices of one respondent parent per household were evaluated via a self-administered, electronic survey that assessed food safety attitudes, handwashing, cleaning, and food preparation behaviors. Most parents considered contamination of food with germs a serious food safety problem (70.0%; Supplemental Table 6). Handwashing before meal preparation and after handling raw animal protein was common; however, no parents reported always washing their hands before touching the refrigerator handle during food preparation. Only 28.6% of parents indicated that they did not wash their hands after electronic device use during food preparation.

All parents reported always washing fresh fruits and vegetables before preparation or consumption. However, 50.0 and 30.0% of parents did report properly washing whole melons and leafy greens, respectively, which was defined as rubbing under running water. No parents who indicated they prepared animal proteins from their raw state reported defrosting raw animal proteins in the refrigerator. Further, 71.4% of parents indicated that they always washed raw poultry, meat, and seafood products before preparation. Washing raw animal proteins in a saline or acidic solution made with citrus juice or vinegar was the preferred method (83.3%) versus washing in water alone. Although all parents reported appropriate contaminated cutting board handling behaviors before reuse, nearly all parents did not own a food thermometer (90.0%).

A high prevalence of cleaning the kitchen counter after food preparation and the kitchen sink after washing the dishes was reported by parents (90.0%, respectively); however, disinfectant or bleach use to clean these surfaces was less common. Further, over half of parents used inappropriate cleaning tools, including dishcloths or dish sponges, to clean the counter (60.0%). Of note, most parents who reported disinfectant or bleach use to clean kitchen counters also reported using an inappropriate cleaning tool (66.7%). Of parents who reported using a dish sponge for dishwashing, most indicated that they replaced their dish sponge once a month or more (71.4%). All dish sponge users reported squeezing out the water from the dish sponge after they finished using it; however, sponge storage conditions were not evaluated.

**Figure 5:**
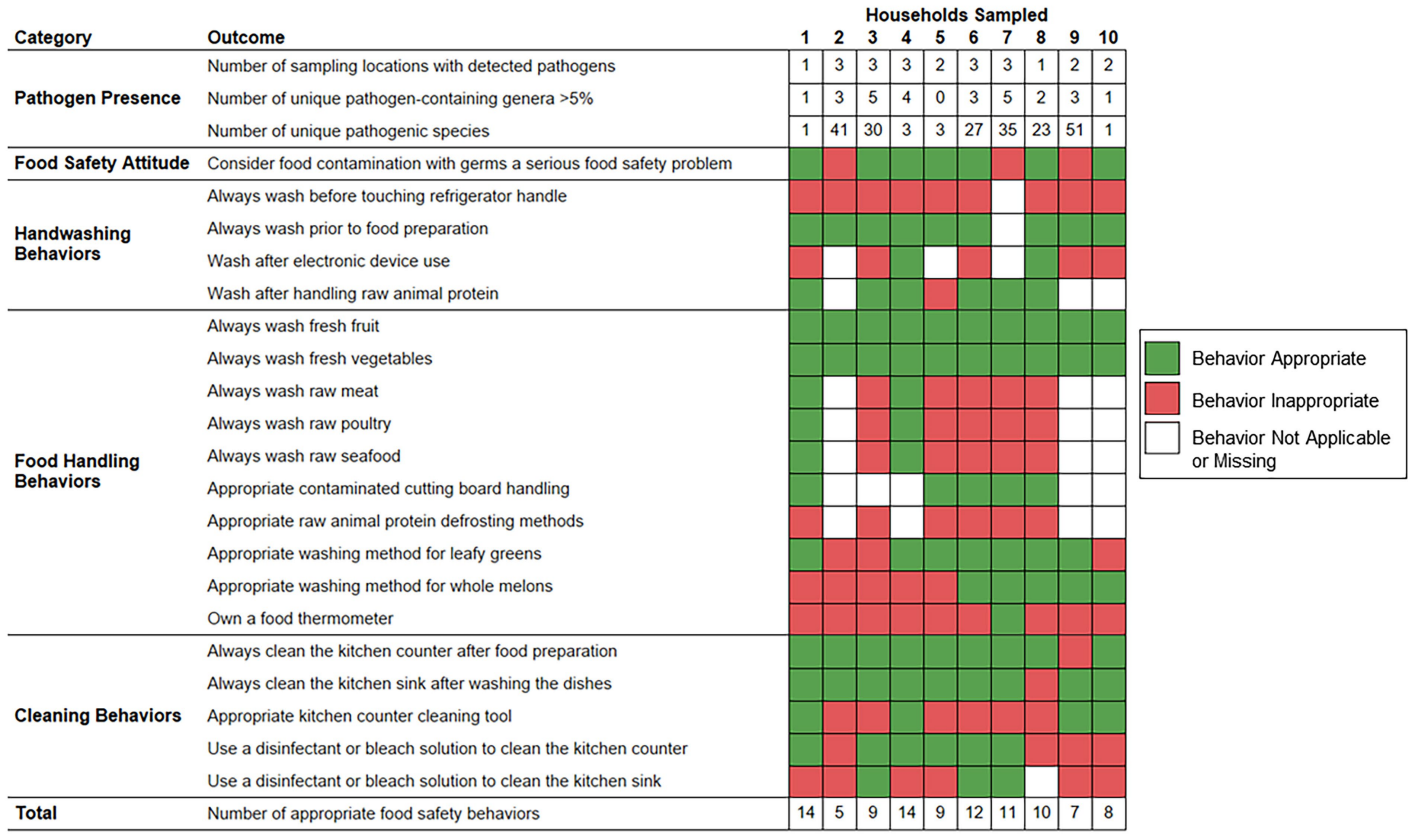
Food safety behaviors reported by respondent parents according to bacterial foodborne pathogen presence detected within predominantly low-income households (*n* = 10), Houston, Texas, 2021. Red color indicates that the respective reported food safety behavior was inappropriate. Green color indicates that the respective reported food safety behavior was appropriate. White color indicates that the food safety behavior was not applicable, or the survey question did not receive a response.

Figure 5 demonstrates the frequency of food safety behaviors reported by each respondent parent according to the numbers of sampling locations positive for foodborne pathogen contamination, unique foodborne pathogen-containing genera, and unique foodborne pathogens identified per household. The number of foodborne pathogens identified was significantly higher among households whose respondent parent did not consider food contamination with germs a serious food safety problem (median: 41.0 species) as opposed to parents that did consider food safety a serious food safety problem (median: 3.0 species; *p* value = 0.0218). The numbers of sampling locations positive for foodborne pathogen contamination, foodborne pathogen-containing genera, and foodborne pathogens per household were not observed to vary significantly according to the demographic groups evaluated or other examined food safety behaviors.

## Discussion

This study evaluated the domestic kitchen bacterial communities of ten predominantly low-income families using targeted 16S rRNA sequencing and the food safety behaviors of one respondent parent per household via a cross-sectional survey. The aim of this research was to ascertain the presence of foodborne pathogens within the households of predominantly low-income families and to examine potential associations between parent demographic and behavioral characteristics and foodborne pathogen contamination across domestic kitchen surfaces. Previous studies have examined the microbiomes of domestic kitchen surfaces and dish sponges (Dunn et al., 2013; Flores et al., 2013; Lax et al., 2014; Cardinale et al., 2017; Jacksch et al., 2020; Møretrø et al., 2021); however, this study was able to taxonomically characterize bacterial communities at the species level and determine the presence of bacterial foodborne pathogens. In addition to comparisons of identified bacterial community diversity and composition across surface types and households, this study also examined the potential associations between respondent parent demographic characteristics and food safety behaviors on foodborne pathogen presence within kitchen microbiomes.

A large degree of variation in microbial community alpha diversity, as measured by Shannon’s diversity index, was observed across sampling locations from the same household and sampling locations of the same type across households. Other studies have found that the highest levels of diversity are located on surfaces that would be touched infrequently or are infrequently cleaned (e.g., interior door trim, stove exhaust fans; Dunn et al., 2013; Flores et al., 2013). The four sampling locations evaluated in this study would likely be used daily by household residents for food preparation or cleaning activities. However, alpha diversity was highest among sink drains, and although nearly all parents reported always cleaning the sink after washing the dishes, the prevalence of disinfectant or bleach use for this purpose was low. The area of the sink underneath the drain trap at the mouth of the drain may also not be cleaned with the same frequency as sink basins, although this study did not evaluate the frequency or method of cleaning these two kitchen surfaces separately. Previously, low alpha diversity has been observed among kitchen sink drains and other sink-associated sites (Flores et al., 2013). However, information on the demographic distribution of household residents or their food safety habits was not provided, which are factors that may impact the diversity levels of kitchen surfaces. For example, in the present study, food safety-related behaviors observed among respondent parents, including the low prevalence of disinfectant use to clean the kitchen sink and high prevalence of both washing raw animal proteins and defrosting raw animal proteins in the sink, may allow the survival of bacterial communities or bacterial migration into sink drain microbiomes.

Of the four sampling locations, a significant difference in microbial community composition was observed between dish sponge and refrigerator handle microbiomes. It has been hypothesized that variation in microbiome composition across kitchen surface types is attributable to the environmental conditions at each location and surface transmission potential (Flores et al., 2013). As refrigerator handles are dry contact surfaces, while dish sponges are moist environments with the potential for high nutrient availability, the variation in microbial community composition observed is likely due to these differing environmental conditions. Transmission potential among refrigerator handles evaluated in this study is also likely high as no parents reported washing their hands before touching the refrigerator handle during food preparation. A previous study of 40 household surface microbiomes found significant differences between the bacterial community composition of nine surface types evaluated, including kitchen cutting boards, kitchen counters, refrigerator shelves, toilet seats, pillowcases, television screens, exterior handles to the main door of each house, upper door trim of the outside of an exterior door, and upper door trim on an interior door (Dunn et al., 2013). The lack of significant variability in taxonomic abundance across the four kitchen surfaces observed in this study may be due, in part, to the limited sample size or the high similarity between kitchen surfaces due to proximity and frequency of use. Significant differences in bacterial community composition across domestic kitchens were also observed among specific households. Humans are important contributors of microbes in their home environment (Flores et al., 2013; Lax et al., 2014), and bacterial communities are highly sensitive to these contributions. Although no significant association was detected between respondent parent demographic characteristics and the composition of bacterial communities in this study, the small sample size and convenience sampling approach may have limited this study’s ability to detect potential differences across demographic groups.

At least one bacterial foodborne pathogen was detected within every domestic kitchen evaluated. Other metataxonomic research has identified foodborne pathogen-containing genera on various kitchen surfaces (Flores et al., 2013). Bacterial human pathogens, including foodborne pathogens, have also been detected on kitchen surfaces via targeted, culture-based methods (Josephson et al., 1997; Ojima et al., 2002; Borrusso and Quinlan, 2017b). One study in Philadelphia found that 45% of households evaluated contained at least one targeted foodborne pathogen (i.e., *S. aureus*, *Listeria*, *Campylobacter jejuni*; Borrusso and Quinlan, 2017b). The difference in pathogen detection rates is likely due to the ability of targeted 16S rRNA sequencing to detect both viable and non-viable bacteria and the limitations of culture-based methods, such as the inability to detect viable but not culturable bacteria. In the present study, families were most frequently at risk of exposure to *E. coli*, *S. aureus*, and *K. pneumoniae* from kitchen surfaces and dish sponges. Similarly, *S. aureus* was the most common pathogen detected (39% of kitchens) of those targeted in the previous study (Borrusso and Quinlan, 2017b). Across the four sampling locations evaluated, dish sponges contained the highest median number of unique foodborne pathogens, while sink drains were most frequently contaminated with foodborne pathogens. Previous culture-based studies have observed the highest levels of coliform contamination among dish sponges and cloths (Josephson et al., 1997; Borrusso and Quinlan, 2017b). One study also found the highest rates of fecal coliform and *E. coli* contamination among kitchen sinks (Borrusso and Quinlan, 2017b). Over half of parents in the present study reported the use of dish sponges to clean the kitchen counter, which due to the high number of observed unique foodborne pathogens identified, is a behavior that may elevate cross-contamination risk.

The assessment of self-reported food safety practices among respondent parents found a high prevalence of several appropriate behaviors that have the potential to reduce cross-contamination and the risk of home-acquired foodborne illness. A similarly high prevalence of these behaviors, including fresh fruit and vegetable washing, handwashing before food preparation and after handling raw animal protein, and appropriate contaminated cutting board handling, has been observed among Women, Infants, and Children (WIC) program clients in Florida (Trepka et al., 2006). Gaps in food safety practices reported among parents included lack of disinfectant or bleach use to clean kitchen surfaces and use of reusable cleaning products. The unavailability of cleaning tools such as paper towels has been observed among low-income households (Borrusso et al., 2015), and financial restrictions may also contribute to the preference for reusable cleaning tools or soap to clean among the parents in the present study. A visual audit of domestic kitchen sanitation paired with microbial sampling found a significantly higher number of coliforms in households that did not contain cleaning products, including disinfectant cleaners and dish soap, in kitchens (Borrusso and Quinlan, 2017b). The absence of cleaning products was also associated with *S. aureus*, *E. coli*, and fecal coliform contamination. The present study similarly found a link between food safety attitude and pathogen presence. Specifically, significantly higher numbers of unique foodborne pathogens were identified within the domestic kitchens of respondent parents who reported they did not consider food contamination with germs to be a serious food safety problem. This data indicates that food safety attitudes may alter domestic bacterial communities and influence the risk of home-acquired foodborne illness among residents.

One limitation of this study is the small number of households evaluated, which may restrict the generalizability of the study results outside of the study population. However, consistent collection practices were used as the same research team obtained all environmental samples. Only four sampling locations were examined in this study, but they were each chosen to represent key sites of bacterial contamination within the built environment, including reservoirs (i.e., sink drains), reservoir disseminators (i.e., dish sponges), and contact surfaces (i.e., refrigerator handles, counters). Although families knew when their household was to be sampled, parents were requested to maintain normal cleaning practices beforehand, and specific sampling locations were not revealed in advance.

Overall, the results of this study indicate that low-income families may be at risk of bacterial foodborne pathogen exposure from contaminated home kitchen surfaces and dish sponges and that food safety attitudes may be associated with pathogen presence. These results also highlight the need for parents to engage in safe food handling and hygiene behaviors to prevent domestic foodborne pathogen exposures and cross-contamination events, which may increase the risk of foodborne illness for themselves and their families. Food safety messaging targeted at parents should describe the risks foodborne pathogens present in the domestic kitchen environment, emphasize the vulnerability of children to foodborne illness, and highlight the possible consequences of improper food safety practices. Future research should include a larger number of households and kitchen sampling locations to further explore potential variation in kitchen surface microbiomes according to household demographic characteristics and food safety behaviors.

## Data availability statement

The datasets presented in this study can be found in online repositories. The names of the repository/repositories and accession number(s) can be found at: NCBI under BioProject PRJNA834026, BioSamples SAMN28027601–SAMN28027640.

## Ethics statement

The studies involving human participants were reviewed and approved by the University of Texas Health Science Center Committee For the Protection of Human Subjects (reference number: HSC-SPH-20-1155; approval date: November 18, 2020). The patients/participants provided their electronic and written informed consent to participate in this study.

## Author contributions

CC collected environmental samples, performed data analysis, and wrote the first draft of the manuscript. CC and JS conducted laboratory experiments and created data visualizations. All authors contributed to the article and approved the submitted version.

## Funding

This research did not receive any specific grant from funding agencies in the public, commercial, or not-for-profit sectors. CD was supported by NIH/NIAID grants R01AI116914 and R01AI150685.

## Conflict of interest

The authors declare that the research was conducted in the absence of any commercial or financial relationships that could be construed as a potential conflict of interest.

## Publisher’s note

All claims expressed in this article are solely those of the authors and do not necessarily represent those of their affiliated organizations, or those of the publisher, the editors and the reviewers. Any product that may be evaluated in this article, or claim that may be made by its manufacturer, is not guaranteed or endorsed by the publisher.
