# Supplementary material for: Evaluation of the kitchen microbiome and food safety behaviors of predominantly low-income families
Source: Front Microbiol. 2022 Sep 29;13:987925. doi: 10.3389/fmicb.2022.987925 (PMC9557297; doi:10.3389/fmicb.2022.987925)
Supplement: Supplementary file 1 [file Data_Sheet_1.docx]

Supplemental Table 1. Demographic characteristics of predominantly low-income parents whose households were environmentally sampled (n = 10), Houston, Texas, 2021.

|  | **Frequency** | **Percentage (%)** |
| --- | --- | --- |
| **Age,** years |  |  |
| ≤35 | 5 | 50.0 |
| >35 | 5 | 50.0 |
| **Sex** |  |  |
| Male | 4 | 40.0 |
| Female | 6 | 60.0 |
| **Race/Ethnicity** |  |  |
| Hispanic | 6 | 60.0 |
| Non-Hispanic | 4 | 40.0 |
| **Education** |  |  |
| Less then high school | 3 | 30.0 |
| High school or more | 7 | 70.0 |
| **Employment** |  |  |
| Employed (full-/part-time) | 7 | 70.0 |
| Homemaker/unemployed | 3 | 30.0 |
| **Primary Household Language** |  |  |
| English or bilingual | 3 | 30.0 |
| Spanish or other language | 7 | 70.0 |
| **Prepares Animal Protein from Raw** *^a^* |  |  |
| Yes | 7 | 77.8 |
| **Food Handling Employment** |  |  |
| Yes | 4 | 40.0 |
| **Individual ≥65 Resides in Home** |  |  |
| Yes | 1 | 10.0 |
| **Pregnant Individual Resides in Home** |  |  |
| Yes | 0 | 0.0 |
| **Pet Ownership** |  |  |
| Yes | 4 | 40.0 |
| *^a^*, Missing values: 1 | | |

Supplemental Table 2. Unique taxa identified and alpha diversity parameters^*^ of the microbial communities present within kitchen sink drains among predominantly low-income households (n = 10), Houston, Texas, 2021.

| **House Identifier** | **Unique Taxonomic Identifications** | **Chao1 Index**  (Index ± Standard Error) | **Shannon’s Diversity Index** | **Simpson’s Index** | **Simpson’s Reciprocal Index** |
| --- | --- | --- | --- | --- | --- |
| 1 | 11 | 14 ± 5 | 1.28 | 0.54 | 2.16 |
| 2 | 184 | 304 ± 42 | 2.53 | 0.83 | 6.05 |
| 3 | 234 | 363 ± 36 | 2.44 | 0.8 | 5.09 |
| 4 | 12 | 14 ± 3 | 2.15 | 0.85 | 6.75 |
| 5 | 5 | 8 ± 5 | 1.04 | 0.59 | 2.46 |
| 6 | 42 | 72 ± 18 | 1.98 | 0.82 | 5.41 |
| 7 | 26 | 77 ± 36 | 2.68 | 0.89 | 8.74 |
| 8 | 2 | 2 ± 1 | 0.69 | 0.5 | 2 |
| 9 | 15 | 20 ± 6 | 1.72 | 0.7 | 3.35 |
| 10 | 12 | 13 ± 2 | 1.95 | 0.81 | 5.4 |
| ^*^, Alpha diversity parameter computations were conducted using the averaged relative abundances or reads assigned to each taxon between replicate samples. | | | | | |

Supplemental Table 3. Unique taxa identified and alpha diversity parameters^*^ of the microbial communities present on disposable dish sponges obtained from predominantly low-income households (n = 10), Houston, Texas, 2021.

| **House** | **Unique Taxonomic Identifications** | **Chao1 Index**  (Index ± Standard Error) | **Shannon’s Diversity Index** | **Simpson’s Index** | **Simpson’s Reciprocal index** |
| --- | --- | --- | --- | --- | --- |
| 1 | 6 | 12 ± 8 | 1.39 | 0.68 | 3.1 |
| 2 | 646 | 976 ± 60 | 3.92 | 0.95 | 20.03 |
| 3 | 346 | 772 ± 101 | 3.89 | 0.96 | 22.52 |
| 4 | 2 | 2 ± 1 | 0.69 | 0.5 | 2 |
| 5 | 5 | 8 ± 5 | 1.42 | 0.74 | 3.85 |
| 6 | 263 | 517 ± 66 | 2.06 | 0.81 | 5.16 |
| 7 | 223 | 410 ± 56 | 2.61 | 0.83 | 5.89 |
| 8 | 271 | 479 ± 54 | 2.82 | 0.82 | 5.53 |
| 9 | 4 | 4 ± 1 | 1.16 | 0.64 | 2.8 |
| 10 | 1 | 1 ± 0 | 0 | 0 | 1 |
| ^*^, Alpha diversity parameter computations were conducted using the averaged relative abundances or reads assigned to each taxon between replicate samples. | | | | | |

Supplemental Table 4. Unique taxa identified and alpha diversity parameters^*^ of the microbial communities present on kitchen counters among predominantly low-income households (n = 10), Houston, Texas, 2021.

| **House** | **Unique Taxonomic Identifications** | **Chao1 Index**  (Index ± Standard Error) | **Shannon’s Diversity Index** | **Simpson’s Index** | **Simpson’s Reciprocal index** |
| --- | --- | --- | --- | --- | --- |
| 1 | 9 | 13 ± 5 | 1.63 | 0.71 | 3.39 |
| 2 | 133 | 202 ± 27 | 2.93 | 0.89 | 9.37 |
| 3 | 7 | 11 ± 5 | 1.82 | 0.82 | 5.58 |
| 4 | 7 | 9 ± 3 | 1.5 | 0.69 | 3.24 |
| 5 | 26 | 61 ± 26 | 1.68 | 0.67 | 3.07 |
| 6 | 2 | 2 ± 1 | 0.69 | 0.5 | 2 |
| 7 | 5 | 7 ± 3 | 1.35 | 0.68 | 3.15 |
| 8 | 8 | 13 ± 6 | 1.64 | 0.74 | 3.84 |
| 9 | 542 | 981 ± 86 | 3.23 | 0.91 | 11.34 |
| 10 | 4 | 7 ± 5 | 1.07 | 0.6 | 2.53 |
| ^*^, Alpha diversity parameter computations were conducted using the averaged relative abundances or reads assigned to each taxon between replicate samples. | | | | | |

Supplemental Table 5. Unique taxa identified and alpha diversity parameters^*^ of the microbial communities present on refrigerator handles among predominantly low-income households (n = 10), Houston, Texas, 2021.

| **House** | **Unique Taxonomic Identifications** | **Chao1 Index**  (Index ± Standard Error) | **Shannon’s Diversity Index** | **Simpson’s Index** | **Simpson’s Reciprocal index** |
| --- | --- | --- | --- | --- | --- |
| 1 | 8 | 13 ± 6 | 1.83 | 0.81 | 5.15 |
| 2 | 3 | 4 ± 2 | 1.04 | 0.62 | 2.67 |
| 3 | 44 | 77 ± 20 | 2.9 | 0.88 | 8.38 |
| 4 | 4 | 7 ± 5 | 1.24 | 0.68 | 3.16 |
| 5 | 7 | 9 ± 3 | 1.34 | 0.65 | 2.88 |
| 6 | 3 | 6 ± 5 | 1.02 | 0.62 | 2.6 |
| 7 | 15 | 27 ± 10 | 2.44 | 0.9 | 9.67 |
| 8 | 2 | 2 ± 1 | 0.69 | 0.5 | 2 |
| 9 | 2 | 3 ± 3 | 0.69 | 0.5 | 2 |
| 10 | 2 | 2 ± 0 | 0.69 | 0.5 | 2 |
| ^*^, Alpha diversity parameter computations were conducted using the averaged relative abundances or reads assigned to each taxon between replicate samples. | | | | | |


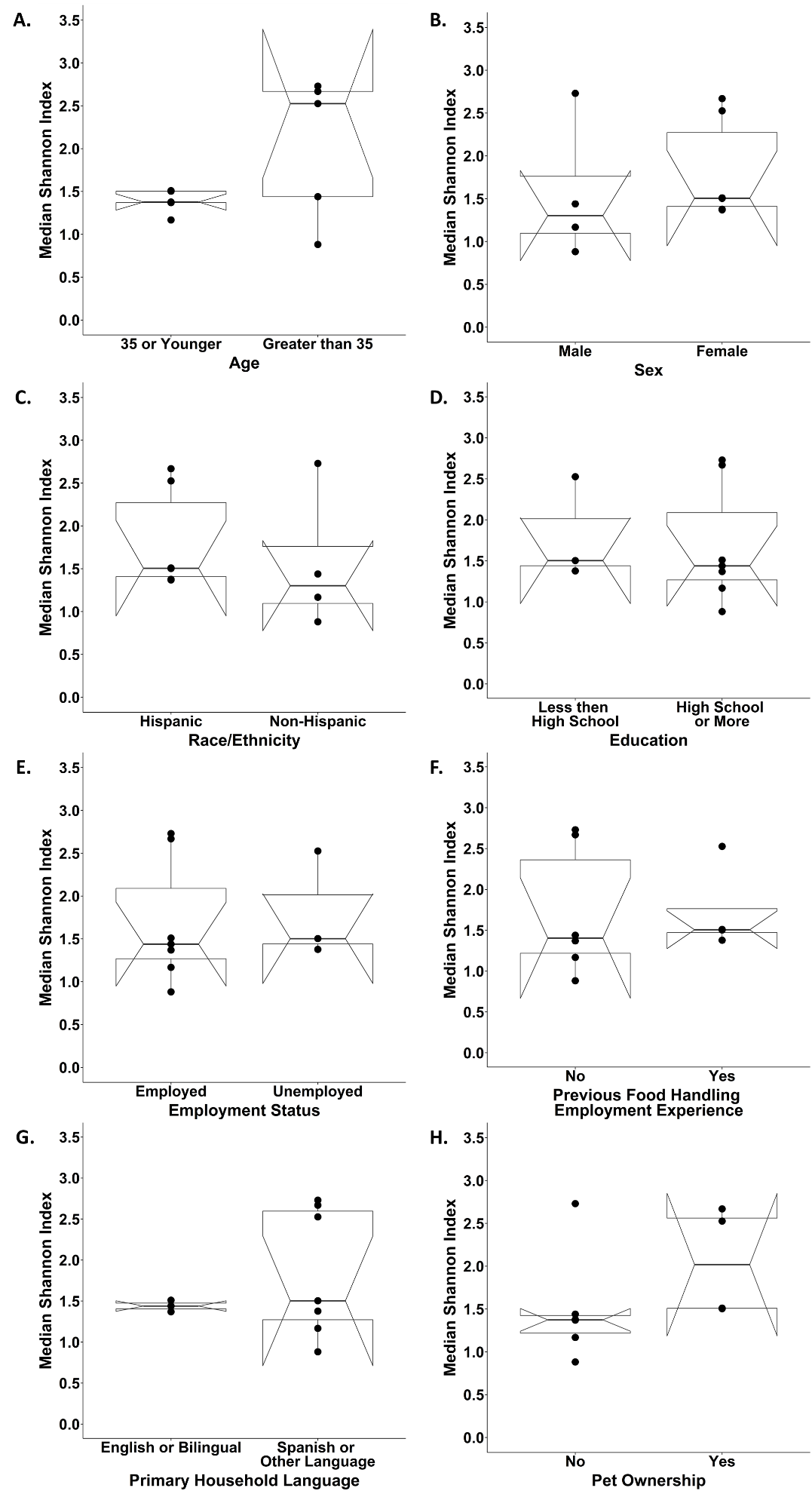


Supplemental Figure 1. The alpha diversity of the microbial communities detected among predominantly low-income households (n = 10) as represented by Shannon’s diversity index according to demographic characteristics including respondent parents’ age (**A**), sex (**B**), race/ethnicity (**C**), education (**D**), employment status (**E**), previous food handling employment experience (**F**), primary household language (**G**), and pet ownership (**H**), Houston, Texas, 2021. Shannon’s diversity index parameter computations were conducted using the averaged relative abundances of taxa between replicate samples.


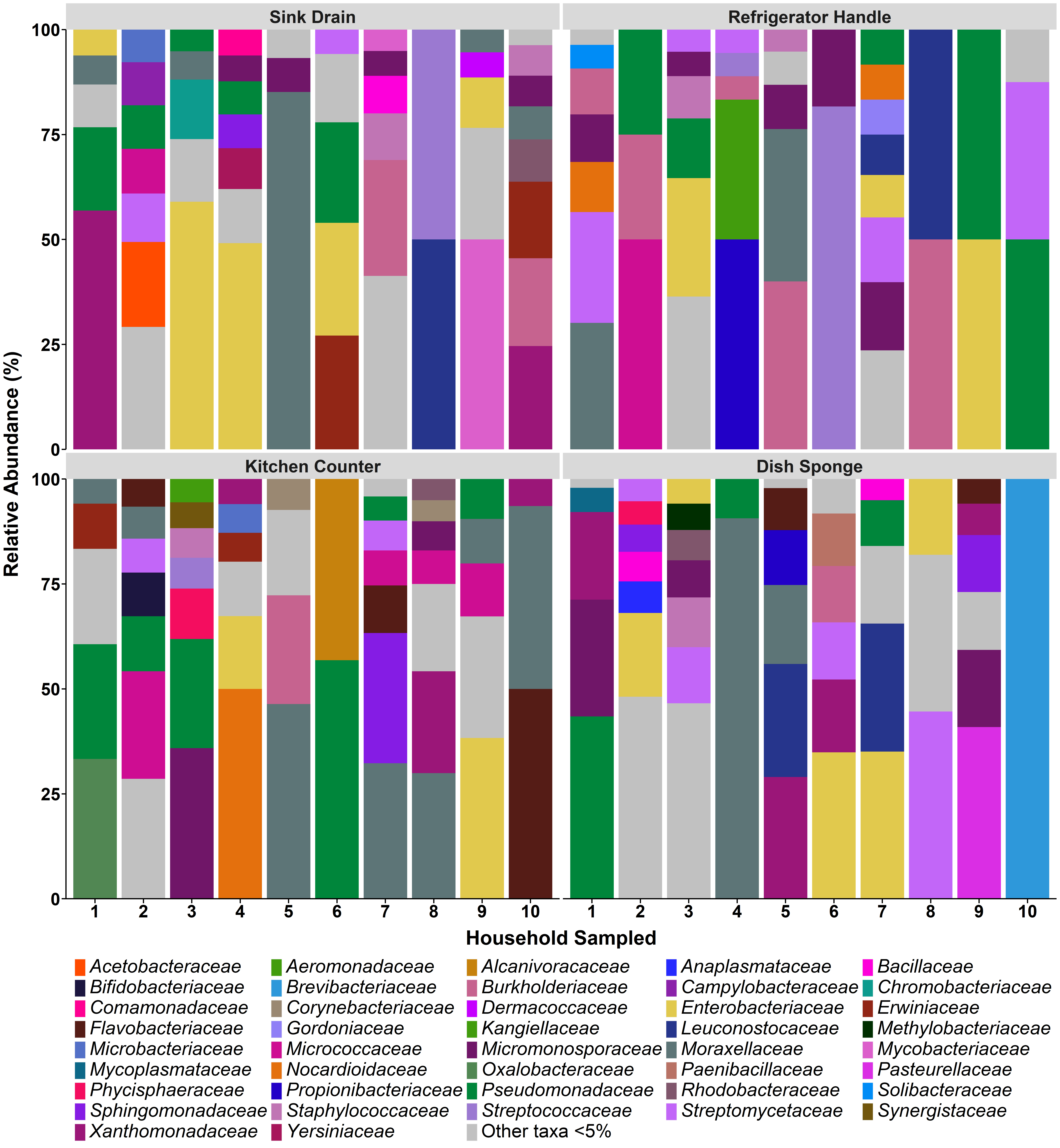


Supplemental Figure 2. The relative abundance of bacterial taxa detected at the family level among four kitchen sampling locations within predominantly low-income households (n = 10), Houston, Texas, 2021. Bars represent averaged relative abundances of taxa between replicate samples.

## Supplemental Table 6. Prevalence of food safety attitude, handwashing, food handling, and cleaning behaviors among predominantly low-income parents whose households were environmentally sampled (n = 10), Houston, Texas, 2021.

|  | **Frequency** *^a^* | **Percentage**  **(%)** |
| --- | --- | --- |
| **Food Safety Attitude** |  |  |
| Consider food contamination with germs a serious food safety problem*^b^* | 7 | 70.0 |
| **Handwashing Behaviors** |  |  |
| Always wash prior to food preparation*^c^* | 9 | 100 |
| Always wash before touching refrigerator handle*^c^* | 0 | 0.0 |
| Wash after electronic device use*^d^* | 2 | 28.6 |
| Wash after handling raw animal protein*^d, e^* | 6 | 85.7 |
| **Food Handling Behaviors** |  |  |
| Appropriate raw animal protein defrosting method*^e, f^* | 0 | 0.0 |
| Always wash raw poultry*^c e^* | 5 | 71.4 |
| Always wash raw meat*^c, e^* | 5 | 71.4 |
| Always wash raw seafood*^c, e^* | 5 | 71.4 |
| Appropriate contaminated cutting board handling*^e, g^* | 5 | 100.0 |
| Own a food thermometer | 1 | 10.0 |
| Always wash fresh fruit*^c^* | 10 | 100.0 |
| Always wash fresh vegetables*^c^* | 10 | 100.0 |
| Appropriate washing method for whole melons*^h^* | 5 | 50.0 |
| Appropriate washing method for leafy greens*^h^* | 7 | 70.0 |
| **Cleaning Behaviors** |  |  |
| Always clean the kitchen counter after food preparation*^c^* | 9 | 90.0 |
| Use a disinfectant or bleach solution to clean the kitchen counter | 6 | 60.0 |
| Appropriate kitchen counter cleaning tool *^i^* | 4 | 40.0 |
| Always clean the kitchen sink after washing the dishes*^c^* | 9 | 90.0 |
| Use a disinfectant or bleach solution to clean the kitchen sink | 3 | 33.3 |
| *^a^*, The sample size for each cross tabulation varies due to applicability of study question or missing data.  *^b^*, Response categories: “serious” or “not serious” food safety problem  *^c^*, Response categories: “always”, “not always”  *^d^*, Response categories: “yes”, “no”  *^e^*, Among those that “always” or “sometimes” prepare raw animal proteins from raw.  *^f^*, Inappropriate defrosting methods included, “on the counter”, “in sink of water”, and “under running water”. Defrosting “in the refrigerator” was considered an appropriate response.  *^g^*, Inappropriate contaminated cutting board handling included “rinsing” or “wiping” before use to prepare other food to be eaten raw for the same meal. “Washing with soap” was considered the appropriate response.  *^h^*, Inappropriate washing methods included, “hold under running water, without rubbing”, “soak in a container of water”, “use a cleaner to wash”. “Rub under running water” was considered the appropriate response.  *^i^*, Inappropriate kitchen counter cleaning tools included dish sponges and dishcloths. Appropriate responses included paper towels or disposable wipes. | | |
